# Styptic Balsam

**Published:** 1854-03

**Authors:** James Warren


### *Styptic Balsam.*

BY JAMES WARREN, M. D.

With a view to a more extensive publicity to the above-named remedy than I have been able to give by private means, I avail myself of your journal for that pupose.

It is nearly thirty years since I commenced the use of this balsam as a styptic, in the various forms of hemorrhage which are within the domain of medical pathology, and with uniform success.

I am satisfied that no remedy now known exerts a more specific power and more speedy relief, especially in hemoptisis, hematemesis, epistaxis and menorrhagia.

It acts both by its sedative power, in diminishing the force of the circulation, and by its astringent qualities, in contact with the bleeding vessels. In the treatment of hemorrhage, neither blood-letting, confinement to the room, suppression of the voice, relaxation from businets, nor other precautions are necessary; nor is any auxiliary treatment required, except, perhaps, a dose of Epsom salts, where there is evidence that blood has been swallowed.

Ordinary exercise in the open air is decidedly preferable to inaction; and wherever there are premonitory symptoms of a return of hemorrhage, it has always exerted a prophylactic power when promptly used; and by this early resort to the remedy, many radical cures have been effected.

The following is the formula and the method of using it:

℞ Acid. Sulphuric. (by weight), ℥v.

Spts. Terebenth. } ℥ij.

Spts. Vin. Rect. *a a* }

Place the acid in a Wedgewood mortar, and add the turpentine slowly, stirring it constantly with the pestle; then add the alcohol in the same manner, and continue stirring it until no more fumes arise, when it may be bottled, and should be stopped with a ground stopper.

It should be prepared from the purest materials; and when done, it should exhibit a dark but clear red color, like dark blood; but if it be a pale, dirty red, it will be unfit for use. The dose is 40 drops, and the method of using it as follows:

Put a tea-spoonful of brown sugar in a common sized tea-cup, and rub in 40 drops of the balsam until it is thoroughly incorporated, and then slowly stir in water until the cup is nearly full, when it should be immediately swallowed.

This dose may be repeated at intervals of an hour, until three or four doses are taken, if necessary; and its use should be discontinued when fresh blood ceases to flow. After standing a few days, a pellicle forms upon the surface of the balsam, which should be broken, and the liquid below it used. It does not deteriate by age, if tightly stopped.—*N. Y. Med. Times.*

